# Biofilm production and virulence traits among extensively drug-resistant and methicillin-resistant *Staphylococcus aureus* from buffalo subclinical mastitis in Bangladesh

**DOI:** 10.1038/s41598-025-17476-2

**Published:** 2025-10-02

**Authors:** Md Shahidur Rahman Chowdhury, Md Mosharof Hosen, Md Hemayet Hossain, Md Rafiqul Islam, Md Bashir Uddin, Md Masudur Rahman, Md Mukter Hossain, Md Mahfujur Rahman

**Affiliations:** 1 Department of Medicine, Sylhet Agricultural University, Sylhet, 3100 Bangladesh; 2 Department of Anatomy and Histology, Sylhet Agricultural University, Sylhet, 3100 Bangladesh; 3 Department of Pathology, Sylhet Agricultural University, Sylhet, 3100 Bangladesh

**Keywords:** Microbiology, Diseases

## Abstract

Methicillin-resistant *Staphylococcus aureus* (MRSA) is a critical pathogen implicated in subclinical mastitis (SCM), a hidden threat to dairy productivity. This study investigated the prevalence, antibiotic resistance profiles, and virulence traits of MRSA from SCM-affected riverine buffaloes in Jamalpur, Bangladesh. A total of 344 milk samples were screened using the California Mastitis Test (CMT) and Modified Whiteside Test (MWST). Among the milk samples, 46.5% were positive for SCM by CMT. Culture, biochemical tests, and PCR confirmed 73 (21.2%) *Staphylococcus* spp., of which 30 (41.1%) were identified as *S. aureus* and 43 (58.9%) as non-aureus staphylococci (NAS). Among the 30 *S. aureus*-positive isolates, 10 (33.3%) were identified as methicillin-resistant *S. aureus* (MRSA), corresponding to a prevalence of 2.9% among the total milk samples. The MRSA isolates exhibited high multidrug resistance, especially to tetracycline (80%) and cefoxitin (80%), and commonly harbored resistance genes such as *tetA* (80%), *aac(3)-iv* (70%), and *sul1* (50%). Virulence genes *hla* (66.7%) and *sea* (50%) were frequently detected, while *icaA* was found in 23.3% of MRSA. Notably, 60% of MRSA isolates were categorized as XDR based on international standard definitions, while 60% were biofilm producers with high MARI values up to 0.92, indicating severe resistance potential. These findings underscore a significant burden of MDR/XDR MRSA with virulence potential in buffalo SCM, posing serious risks to animal and public health.

**Supplementary Information:**

The online version contains supplementary material available at 10.1038/s41598-025-17476-2.

## Introduction

Buffalo rearing is an important part of rural farming systems in the world, making a significant contribution to the world’s dairy and meat supply^1^. Although buffaloes possess a high degree of adaptability to diverse ecological environments and an innate capacity to withstand climatic stresses, their use has yet to be fully exploited in most countries, particularly in Bangladesh. Subclinical mastitis (SCM) is a significant barrier to the achievement of the highest productive efficiency, yet economically significant intra-mammary infection threatens milk quality, udder health, and farm profitability^2^. Two common buffalo ecotypes, the swamp and riverine, are found in Bangladesh under various agro-ecological circumstances^3^. Riverine buffaloes thrive in riverine areas like Jamalpur, while swamp buffaloes are mainly found in the flooded haor regions of northeastern Bangladesh^3^. In Bangladesh, dairy products derived from buffalo milk are gaining popularity and have the potential to contribute significantly to the dairy sector. However, buffalo farming and milk production remain underdeveloped and insufficiently expanded across the country. The Buffalo Development Project has been initiated to develop expansion and improve production systems at the national level. Such initiatives, however, are generally faced with vigorous challenges due to the widespread, subclinical occurrence of mastitis, which gradually undermines herd productivity.

Over the past half-century, buffalo farming has seen tremendous expansion across Asia, producing about 13% of global milk output^4^. In the 2023–2024 fiscal year, Bangladesh had a ruminant population of 575.57 lakh (57.557 million), producing 150.44 lakh (15.044 million) metric tons of milk, with buffalo numbers increasing to 15.24 lakh (1.524 million)^5^. Even with this growth, however, mastitis has remained one of the most persistent and economically draining diseases among dairy farming, significantly reducing the volume and value of milk. The disease is responsible for tremendous economic losses, primarily in the form of therapy and lost productivity, with an estimated annual loss of $70 per infected buffalo, comprising 55% for therapeutic intervention and 16% for lost milk production^6,7^. Mastitis also presents in its clinical (CM) and SCM forms where SCM is more frequent and insidious. Occurring 15 to 40 times more frequently than CM, SCM lacks apparent clinical symptoms so that it can barely be noticed and treated, hence leading to extended milk production loss and long-term economic consequences^8^. SCM is caused by multiple factors, including host immunity, nutrition, pathogen virulence, and poor management practices^9^. Among the pathogens, *S. aureus* especially MRSA is highly significant due to its prevalence, chronic and recurrent nature, as well as impact on both animal and public health^10^. Milk-borne MRSA is an emerging threat in dairy production due to its biofilm-forming ability, which enhances resistance to antibiotics and sanitizers. Biofilm-producing MRSA has been found in raw, pasteurized, and UHT (Ultra Heat Treatment) milk, with higher rates in pasteurized milk^11^. Genes like *icaA* and *icaD* aid its persistence in dairy settings^12^. Interventions such as improving hygiene or introducing lactic acid bacteria show promise in reducing MRSA biofilms.

As a widely circulating zoonotic agent, *S. aureus* causes damage to both humans and animals, thus generating huge health problems in most contributing sectors^13,14^. Moreover, Methicillin-resistant strains (MRSA), also referred to as “superbugs,” cause serious hospital-acquired infections, which lead to higher mortality and higher health expenditure^14,15^. In livestock, particularly dairy buffalo and cattle, *S. aureus* is a primary etiologic cause of mastitis and the primary contributor to SCM burden^16–21^. It has been implicated in approximately one-third of SCM cases, a pathogenicity that is most affected by its ability to produce a range of virulence factors^19,20^. The most prominent characteristic among the features of *S. aureus* is its high effectiveness in becoming resistant to extremely wide ranges of antimicrobial drugs because it carries a wide variety of antimicrobial resistance genes, often on mobile genetic elements. This allows the bacterium to quickly adapt and thrive even when antibiotics are present^22^. Over time, this has therefore led to the emergence of multidrug-resistant *S. aureus* (MDR-SA) strains, which are resistant to multiple classes of antibiotics and hence have narrow therapeutic options^14,23,24^. According to internationally recognized criteria set forth by the European Centre for Disease Prevention and Control (ECDC) and the Centers for Disease Control and Prevention (CDC), MDR *S. aureus* is defined as being non-susceptible to at least one agent in three or more antimicrobial classes, while extensively drug-resistant (XDR) *S. aureus* refers to strains that are non-susceptible to at least one agent in all but two or fewer antimicrobial classes^25^. The continued emergence of such drug-resistant strains, particularly those falling under the XDR category, poses a grave and growing threat to both animal and human health, particularly within dairy settings due to heightened zoonotic risk; thus, comprehensive genomic surveillance, antimicrobial stewardship, and targeted prevention are vital for safeguarding well-being^26–28^.

To address the emerging issue of AMR, planned and prudent treatment is essential for controlling SCM in buffaloes. But the rapid increase of antibiotic resistance, fueled by inappropriate usage, now gravely imperils the effective treatment of buffalo mastitis, escalating costs and fostering resilient pathogens that threaten both animal welfare and public health^29^. In particular, treatment of mastitis induced by *S. aureus* is most commonly marred by the MRSA strain^30^.

While numerous studies in Bangladesh have investigated the prevalence, antimicrobial susceptibility patterns, and risk factors associated with *S. aureus* in bovine SCM; data pertaining specifically to buffaloes, especially regarding MRSA, remain scarce. This gap is particularly notable in regions like Jamalpur, Bangladesh, which host large populations of riverine buffaloes, underscoring a broader need for species-specific surveillance across endemic areas. The objective of this study was to investigate the prevalence, AMR profile, biofilm-forming ability, and virulence of multidrug-resistant and extensively drug-resistant MRSA strains isolated from buffaloes with subclinical mastitis in Bangladesh.

## Methods

### Ethical consideration

This study was approved by the Animal Experimentation and Ethics Committee (AEEC, SAU) of Sylhet Agricultural University under the approval number AUP2023001. All experimental procedures were performed by qualified personnel in full accordance with the university’s ethical standards and regulations. Animal welfare was given the highest priority, and the well-being of all animals was rigorously monitored and maintained throughout the study.

### Study design, location, and sampling strategy

**Fig. 1:**
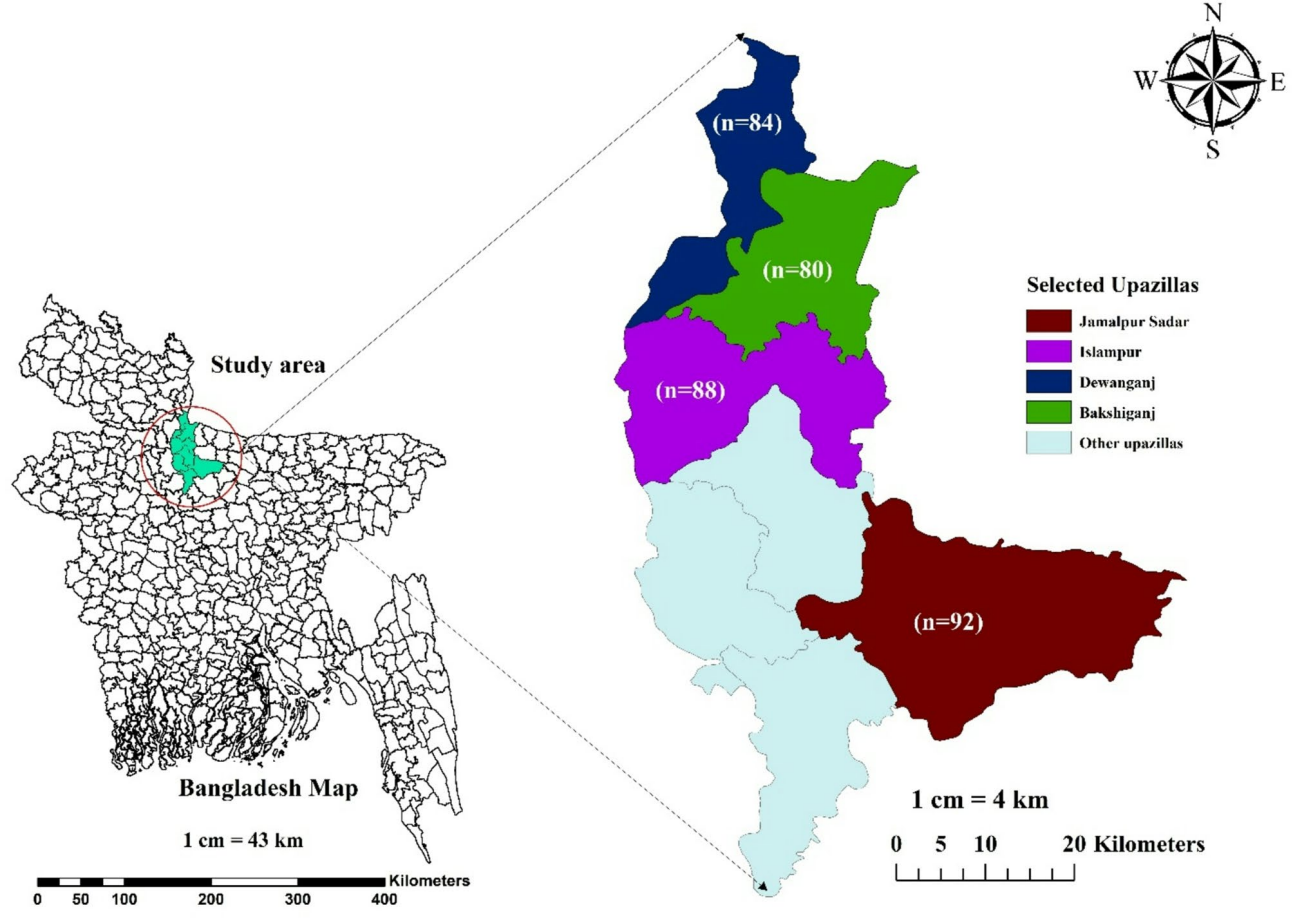
Map of the study area showing the selected upazilas of Jamalpur district, Bangladesh, along with the number of samples collected from each upazila. The map was generated using ArcMap 10.8.

A cross-sectional study was conducted in four Upazilas of Jamalpur District in Bangladesh, which are primarily buffalo-populated areas (Fig. 1). These Upazilas included Bakshiganj, Dewanganj, Islampur, and Jamalpur Sadar. The study population required to estimate prevalence was calculated using a standard Eqs. 3^1,32^.

$$\:\varvec{n}=\frac{{\varvec{Z}}^{2}\times\:\varvec{P}\varvec{e}\varvec{x}\varvec{p}\times\:\left(1-\varvec{P}\varvec{e}\varvec{x}\varvec{p}\right)}{{\varvec{d}}^{2}}$$

[Where, n = Desired sample size; Z = 1.96 for 95% CI; P_exp_ = 0.323, Expected prevalence (32.3%); d = 0.05]^33^.

**Fig. 2:**
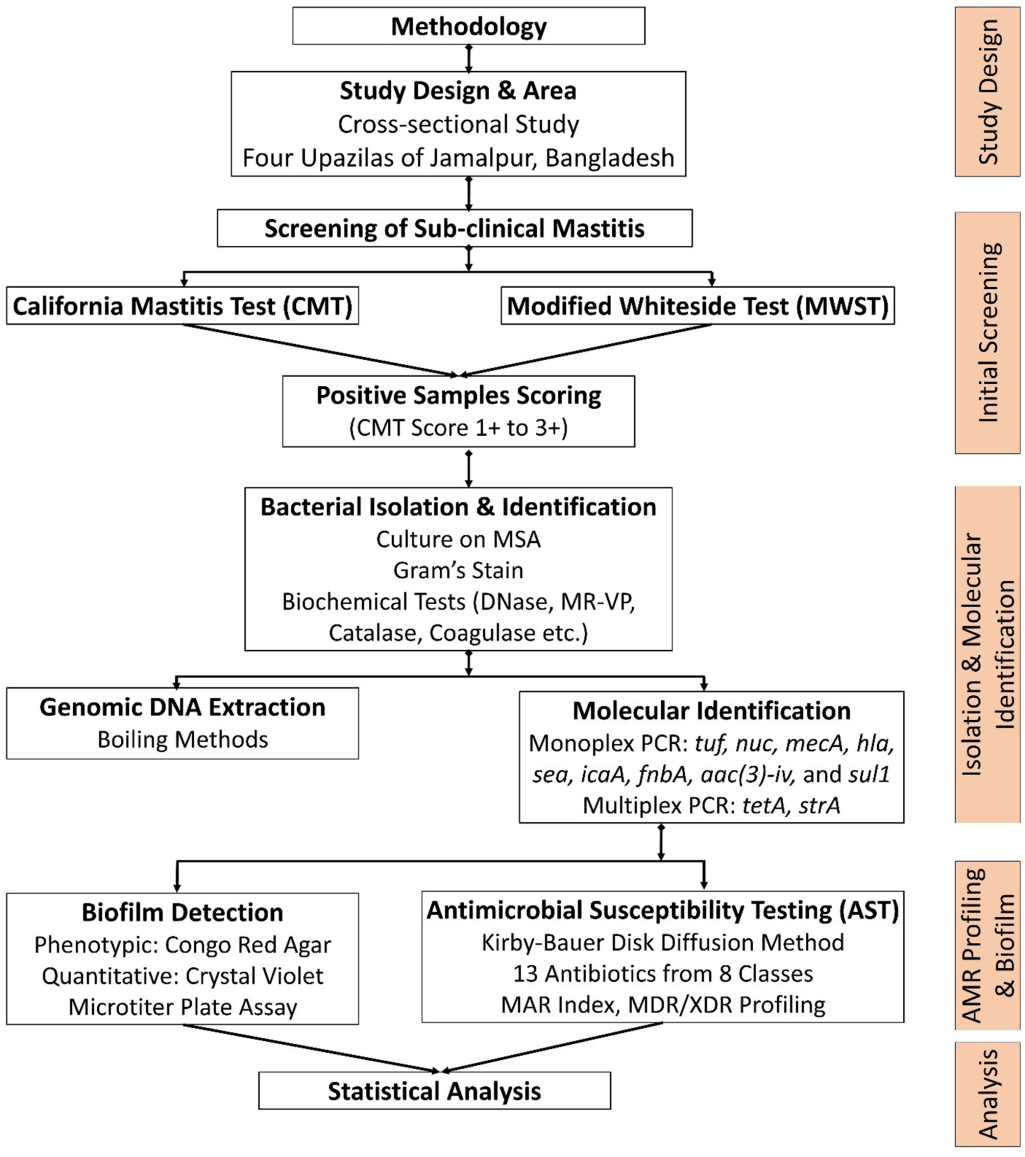
The flow chart of the detailed methodology used in this study.

Based on the calculations, a minimum of 336 quarter milk samples were required to conduct the test. The study proceeded with the collection of 344 quarter milk samples from 86 buffaloes to determine both quarter-level and animal-level prevalence. The samples were collected using a random-cluster sampling strategy from June 2023 to May 2024. A detailed flow chart outlining the methodology employed in this study is presented in Fig. 2.

### Preliminary detection of SCM

The National Mastitis Council’s (NMC) recommendations were followed when aseptically collecting milk samples from each quarter of buffaloes that appeared to be in good health^34^. The Modified Whiteside test (MWST), outlined by Emon et al.^35^ along with the California Mastitis Test (CMT)^21^ were utilized as widely used field-based excellent screening tools that are practical, cost-effective, and rapid methods for early detection and on-farm monitoring of subclinical mastitis (SCM)^21^. Following aseptic milk collection, the samples were combined on a CMT paddle with a CMT reagent in a predetermined ratio. After giving the mixture a gentle whirl, the reaction was monitored for consistency changes. The CMT results are interpreted based on the degree of gel formation that occurs when milk is mixed with the reagent. A CMT grade of 0 (Negative) indicates that the mixture remains liquid and similar to normal milk, suggesting no infection and a healthy udder with a normal SCC^36^. A grade of 1+ (Trace, possible early SCM) shows slight slime formation or a faint gel, which may indicate a mild or early-stage infection with a slightly elevated SCC and warrants monitoring. A grade of 2+ (Distinct Positive, likely SCM) is characterized by moderate gel formation, where the mixture thickens noticeably, suggesting a moderate level of infection and a significantly increased SCC, indicative of subclinical mastitis. Finally, a grade of 3+ (Strong Positive, Definite SCM) is marked by a thick gel that clumps and adheres to the paddle, reflecting a high SCC and confirming a severe form of subclinical mastitis with marked inflammation in the udder^33^. The primarily positive milk samples (CMT graded as 1+, 2+, 3+) underwent pre-enrichment immediately in Trypticase Soya Broth (Oxoid, UK) at a 1:10 dilution, just after initial detection on the same day. Subsequent incubation of these cultures was at 37 °C for around 24 h.

### Isolation and identification of pathogens

Mannitol Salt Agar (Oxoid, UK) was used to detect *S. aureus* in accordance with NMC, USA recommendations and procedures followed by Eidaroos et al.^37^. The streak plate method was applied and incubated at 37 °C for 18 to 24 hours^38^. Biochemical tests confirmed the presence of *S. aureus* with positive results for gram staining, DNase, Methyl Red (MR) test and Voges-Proskauer (VP), catalase, coagulase, citrate utilization, methyl red, and urease tests, while negative results were obtained for motility, indole, gas production, and the triple sugar iron test^35,39^. Positive samples were prepared for genomic DNA extraction and polymerase chain reaction (PCR).

### Genomic DNA extraction and molecular detection of pathogens, virulence genes, and ARGs

**Fig. 3:**
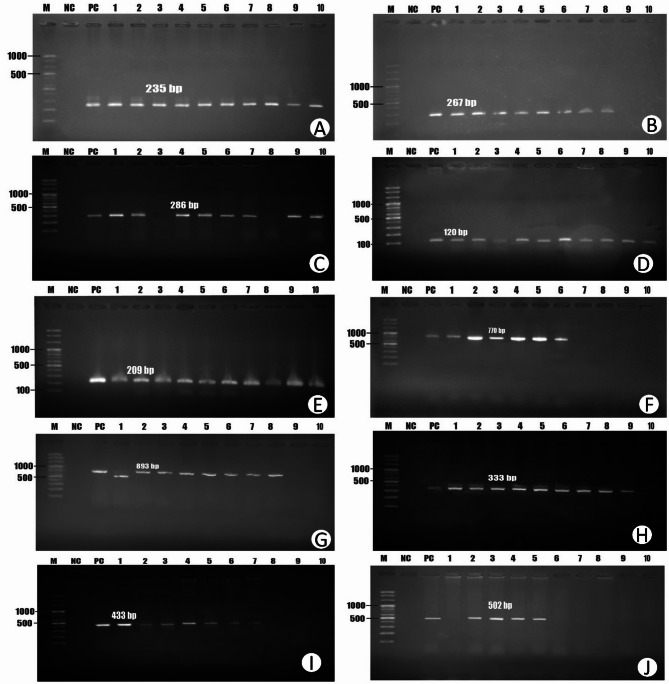
PCR amplification of different genes. (**A**) *tuf* gene (235 bp); (**B**) *nuc* gene (267 bp); (**C**) *mecA* (286 bp); (**D**) *sea* gene (120 bp); (**E**) *hla* gene (209 bp); (**F**) *icaA* gene (770 bp); (**G**) *strA* gene (983 bp); (**H**) *aac(3)-iv* gene (333 bp); (**I**) *sul1* gene (433 bp); (**J**) *tetA* gene (502 bp). M: Marker 100 bp DNA ladder; NC: negative control; PC: positive control.

The DNA from *S. aureus* was extracted using the boiling method as described by Aldous et al.^40^. The monoplex PCR assays targeted the *tuf*,* nuc*,* mecA*,* aac(3)-iv*,* sul1*,* hla*,* sea*,* icaA*, and *fnbA* gene. The multiplex PCR assays focused on the *tetA*, and *strA* gene. In this study, the *tuf* and *nuc* genes are essential for *Staphylococcus aureus* identification and basic cellular functions, with *nuc* also contributing to virulence and biofilm degradation. Resistance to antibiotics is conferred by *mecA* (methicillin), *aac(3)-iv* (aminoglycosides), *sul1* (sulfonamides), and the *tetA* and *strA/strB* genes (tetracycline and streptomycin, respectively), often through efflux pumps or enzymatic modification. Virulence and biofilm formation are significantly mediated by *hla* (alpha-hemolysin), *sea* (enterotoxin A), *icaA* (biofilm polysaccharide synthesis), and *fnbA (*fibronectin binding and adhesion), all crucial for pathogenesis and host interaction. However, the molecular detection of *Staphylococcus* species, virulence genes, and antibiotic resistance genes (ARGs) was conducted using different PCR techniques. For the detection of the *Staphylococcus* genus and *S. aureus*, monoplex PCR targeted the *tuf* gene and the *nuc* gene, respectively, utilizing reagents from SFC Probes Ltd. (Gyeonggi, Korea). Here, the *tuf* gene, responsible for producing the elongation factor Tu, is a conserved gene found across all Staphylococcus species. Its stability and presence in all staphylococci make it a reliable tool for confirming that a bacterial isolate belongs to the Staphylococcus genus^41,42^. In contrast, the *nuc* gene, which codes for a heat-resistant nuclease, is specific to *Staphylococcus aureus*^43,44^. Additionally, more separate monoplex PCRs were employed to identify MRSA by targeting the *mecA* gene as well as antibiotic-resistant genes (*aac(3)-iv* and *sul1) and* virulence genes *(hla*,* sea*,* icaA*, and *fnbA)*. The components of the PCR reaction mixture and the thermal cycling parameters for the multiplex and monoplex assays are listed in Table S1. Using either 1.8% or 1.5% standard agarose gels, amplified PCR products were subjected to gel electrophoresis for analysis. To verify the anticipated fragment sizes, a 100 bp plus DNA ladder was used. All the detected positive genes are illustrated in Fig. 3. Sequences of primers utilized to amplify each target gene are detailed in Table 1.

**Table 1:** Primer details used for monoplex and multiplex PCR for the detection of *Staphylococcus* spp., *S. aureus*, methicillin resistance (*mecA*), virulence genes, and selected antimicrobial resistance genes.

Type of PCR	Primers (Gene)	Targeted genes/Organism	Primer sequence (5’– 3’)	Amplicon size(bp)	Annealing temp. (°C)	References
Monoplex	*tuf*	Genus *Staphylococcus*	F: GAAGAATTATTAGAATTAGT R: GTGATTGAGAATACGTCCTCAAC	235	56	^55^
	*nuc*	*Staphylococcus aureus*	F: GCGATTGATGGTGATACGGTT R: AGCCAAGCCTTGACGAACTAAAGC	267	50	^55^
	*mecA*	Methicillin resistant *Staphylococcus aureus*	F: TGCTATCCACCCTCAAACAGG R: AACGTTGTAACCACCCCAAGA	286	55	^55^
	*hla*	*hla*	F-CTGATTACTATCCAAGAAATTCGATTG R-CTTTCCAGCCTACTTTTTTATCAGT	209	63	^71^
	*sea*	*sea*	F-TTGGAAACGGTTAAAACGAA R-GAACCTTCCCATCAAAAACA	120	63	
	*icaA*	*icaA*	F-GATTATGTAATGTGCTTGGA R-ACTACTGCTGCGTTAATAAT	770	66	
	*fnbA*	*fnbA*	F-GCGGAGATCAAAGACAA R-CCATCTATAGCTGTGTGG	1279	58	
	*aac(3)-iv*	Gentamicin resistant gene	F: AGTTGACCCAGGGCTGTCGC R: GTGTGCTGCTGGTCCACAGC	333	58	^77^
	*sul1*	Sulphonamide resistant gene	F: CGGCGTGGGCTACCTGAACG R: GCCGATCGCGTGAAGTTCCG	433	66	
Multiplex	*tetA*	Tetracycline resistant gene	F: GGCGGTCTTCTTCATCATGC R: CGGCAGGCAGAGCAAGTAGA	502	63	^77^
	*strA*	Streptomycin resistant gene	F: ATGGTGGACCCTAAAACTCT R: CGTCTAGGATCGAGACAAAG	893	63	

### Detection of biofilm producers

To evaluate the biofilm-forming ability of the bacterial isolates, a Congo Red Agar (CRA) assay was conducted, with modifications based on the protocol described by Freeman et al.^45^. The CRA medium was formulated by enriching Brain Heart Infusion (BHI) agar with 0.8 g/L Congo Red dye and 36 g/L sucrose (HiMedia Laboratories Pvt. Ltd., Mumbai, India). Freshly prepared CRA plates were inoculated by streaking the test isolates and incubated aerobically at 37 °C for 24–48 h. Biofilm production was inferred from the phenotypic appearance of the colonies. Isolates that developed dry, black, and rough colonies with a crystalline consistency were interpreted as strong biofilm producers. In contrast, colonies that appeared smooth, red, or lacked pigmentation were identified as weak or non-biofilm formers. This phenotypic approach provided a rapid and cost-effective means of discriminating between high and low biofilm-producing strains. The quantitative determination of biofilm-forming potential among bacterial isolates was carried out using the Crystal Violet Microtiter Plate (CVMP) assay, with methodological adaptations based on Kouidhi et al.^46^. The whole procedure was conducted according to the standard protocol as described by Chowdhury et al.^47^. Bacterial cultures were overnight adjusted to 0.5 McFarland standard and diluted 1:100 in Tryptic Soy Broth (TSB) with 1% glucose for enhanced biofilm formation. A 96-well flat-bottom microtiter plate was used, in which 200 µL of the diluted bacterial suspension was inoculated in each test well. Negative control wells had only sterile TSB with 1% glucose. The plate was kept at 37 °C for incubation under static conditions for 24 h. Non-adherent cells were removed after incubation by washing the wells gently three times with phosphate-buffered saline (PBS, pH 7.2). The adhered biofilm was then stained using 0.1% crystal violet for 15 min at room temperature. Excess stain was washed off by washing the wells three times with sterile distilled water, and the bound dye was solubilized with 95% ethanol. The absorbance was read at 570 nm (OD570) in a microplate reader, and biofilm production was categorized based on OD570 values, with non-biofilm (OD ≤ 0.2), weak (0.2 < OD ≤ 0.4), moderate (0.4 < OD ≤ 0.6), and strong (OD > 0.6) biofilm producers identified accordingly.

### Antimicrobial susceptibility testing

Antimicrobial Susceptibility testing (AST) was executed employing the Kirby-Bauer disk diffusion method on Mueller-Hinton agar (MHA) plate, following the guidelines followed by the Clinical and Laboratory Standards Institute (CLSI)^48,49^. We tested a total of 13 antibiotics comprising 8 distinct groups according to CLSI guidelines 2020, as shown in Table S2, which are most commonly used in Bangladesh, especially for treating mastitis and other bacterial infections in large ruminants, including buffaloes. To meet the 0.5 McFarland standard, freshly isolated colonies of bacteria were suspended in 5 mL of regular saline. After spreading this suspension evenly onto MHA plates, it was incubated for 18 to 24 h at 35 to 37 °C. Following incubation, CLSI breakpoints were compared to the inhibition zone diameters, which were measured in millimeters (mm). To ensure precision and repeatability, each assay was carried out in triplicate^50^. The Multiple Antibiotic Resistance (MAR) index was ascertained following the protocol delineated by Naser et al.^32^ Utilizing the equation: MAR = (Number of antibiotics exhibiting resistance by an isolate)/(Total number of antibiotics subjected to testing). MAR index values spanned from 0 to 1, where values closer to zero indicated heightened susceptibility, while those nearing 1 signified pronounced resistance. A MAR index of 0.20 or higher suggests a significant presence of bacterial contamination or resistance, which could pose a public health concern. In addition, non-susceptibility to at least one agent in three classes of antibiotics or three antimicrobial categories is designated as MDR^25^ while non-susceptibility to at least one agent in all but 2 or fewer antimicrobial categories is termed as XDR^51^.

### Geo-spatial mapping and plot

In this study, the map of the study area was generated using ArcMap 10.8. The shapefile was initially obtained from DIVA-GIS, and the selected upazilas relevant to the study were subsequently visualized. All graphical representations and plots were created using GraphPad Prism 8.4 and Chiplot (https://www.chiplot.online/).

### Statistical analysis

All data were initially recorded and organized in Microsoft Excel. To assess variations in the prevalence across different levels of measured variables, the chi-square test was performed. Descriptive statistics were also employed, and confidence intervals were calculated using the binomial exact test. All statistical analyses were conducted using SPSS version 26, with a significance level set at *p* < 0.05.

## Results

### Prevalence of SCM based on CMT scores

The prevalence of SCM varied among the regions tested. Out of 344 quarter milk samples, Bakshiganj Upazila found 41 positive samples (51.3%), with the majority of scoring 1 + to 3+. Islampur Upazila showed a relatively lower prevalence with 30 positive quarters (34.1%). In contrast, Dewanganj had the highest rate, with 46 positive quarters (54.8%), primarily scoring 1 + or 2 + and Jamalpur Sadar recorded 43 positives (46.7%), mostly with mild (1+) scores (Table 2).

**Table 2:** California mastitis test (CMT) results and prevalence of subclinical mastitis in dairy buffaloes across different upazilas of Jamalpur district.

Name of the test	Total number of samples	Upazilla	Total quarter	Score				No of sample positive	Prevalence (%)
				0 (-ve)	1+	2+	3+		
CMT	344	Bakshiganj	80	39 (48.8)	11 (13.8)	18 (22.5)	12 (15.0)	41	51.3%
		Islampur	88	58 (65.9)	15 (17.0)	9 (10.2)	6 (6.8)	30	34.1%
		Dewanganj	84	38 (45.2)	26 (31.0)	17 (20.2)	3 (3.6)	46	54.8%
		Jamalpur	92	49 (53.3)	33 (35.9)	8 (8.7)	2 (2.2)	43	46.7%

At the animal level, the highest prevalence was observed in Dewanganj (71.4%; 95% CI: 52.1–90.8%), followed by Bakshiganj (65.0%), Jamalpur (52.2%), and Islampur (36.4%) with an overall rate of 55.8% (95% CI: 45.3–66.3%). Quarter-level prevalence followed a similar trend, highest in Dewanganj (54.8%) and lowest in Islampur (34.1%), with an overall rate of 46.5% (95% CI: 41.2–51.8%). No statistically significant difference (*p* > 0.05) was found among the Upazilas (Table 3).

**Table 3:** Animal and quarter level prevalence based on CMT result.

Attributes	Level of measures (*N*)	Proportion; *n* (%)	95% CI	*p*-value
Bakshiganj	AL (20)	13(65)	(44.1% – 85.9%)	0.3938
	QL (80)	41(51.3)	(40.3% – 62.2%)	
Islampur	AL (22)	8(36.4)	(16.3% – 56.5%)	1.0000
	QL (88)	30(34.1)	(24.2% – 44.0%)	
Dewanganj	AL (21)	15(71.4)	(52.1% – 90.8%)	0.2554
	QL (84)	46(54.8)	(44.1% – 65.4%)	
Jamalpur	AL (23)	12(52.2)	(31.8% – 72.6%)	0.8155
	QL (92)	43(46.7)	(36.5% – 56.9%)	
Overall	AL (86)	48(55.8)	(45.3% – 66.3%)	0.1546
	QL (344)	160(46.5)	(41.2% – 51.8%)	

AL: Animal level, QL: Quarter level; N: Number of Sample tested; n: Number of sample positive; CI: Confidence Interval.

### Identification of the pathogen through different methods

A combination of screening (MWST, CMT), culture, biochemical, and molecular methods (PCR) was used to identify *Staphylococcus* spp., *S. aureus*, non-aureus *staphylococci* (NAS), methicillin-resistant *S. aureus* (MRSA), and methicillin-susceptible *S. aureus* (MSSA) from milk samples of different buffalo quarters (LF, LR, RF, RR) as shown in Table 4.

**Table 4:** Distribution of subclinical mastitis screening results and molecular identification of *Staphylococcus* species in different quarters of dairy buffaloes.

Quarter	Screening Test (*n*, %)			PCR (*n*, %)					
	MWST	CMT	C & B for Staphylococcus	Staphylococcus spp.	S. aureus	NAS		MRSA	MSSA
LF	45(52.3)	45(52.3)	27(31.4)	25(29.1)	9(36.0)	16(64.0)		4(44.4)	5(55.6)
LR	43(50.0)	41(47.7)	21(24.4)	20(23.3)	8(40.0)	12(60.0)		2(25.0)	6(75.0)
RF	36(41.9)	36(41.9)	14(16.3)	14(16.3)	5(35.7)	9(64.3)		3(60.0)	2(40.0)
RR	39(45.3)	38(44.2)	15(17.4)	14(16.3)	8(57.1)	6(42.9)		1(12.5)	7(87.5)
**Total**	**163(47.4)**	**160(46.5)**	**77(22.4)**	**73(21.2)**	**30(41.1)**	**43(58.9)**		**10(33.3)**	**20(66.7)**

Modified White Side Test (MWST), California Mastitis Test (CMT), and culture & biochemical (C & B) tests; non-aureus staphylococci (NAS), methicillin-resistant *S. aureus* (MRSA), and methicillin-susceptible *S. aureus* (MSSA) across left front (LF), left rear (LR), right front (RF), and right rear (RR) quarters. Results are expressed as number of positive samples (n) and corresponding percentages (%).

Out of 344 quarter samples examined, MWST and CMT identified 163 (47.4%) and 160 (46.5%) quarters as subclinical mastitis positive, respectively. Culture and biochemical tests confirmed 77 (22.4%) staphylococcal isolates, of which PCR confirmed 73 (21.2%) as *Staphylococcus* spp. Among the *Staphylococcus* spp., 30 (41.1%) were confirmed as *S. aureus*, while 43 (58.9%) were non-aureus staphylococci (NAS). The *S. aureus* isolates were identified based on characteristic colony morphology on Mannitol Salt Agar and further supported by positive catalase and DNase activity, along with a distinct biochemical profile as described in the Methods.

Further characterization revealed that 10 (33.3%) of the *S. aureus* isolates were methicillin-resistant (MRSA) and 20 (66.7%) were methicillin-susceptible (MSSA). The highest detection of *S. aureus* (57.1%) was found in the RR quarters, whereas NAS was most frequently isolated from the LF and LR quarters. Notably, MRSA was identified in all quarters, with the highest proportion in the RF quarter (60.0%).

### Antibiogram profile of MRSA and distribution of virulence and antibiotic-resistant genes

**Fig. 4:**
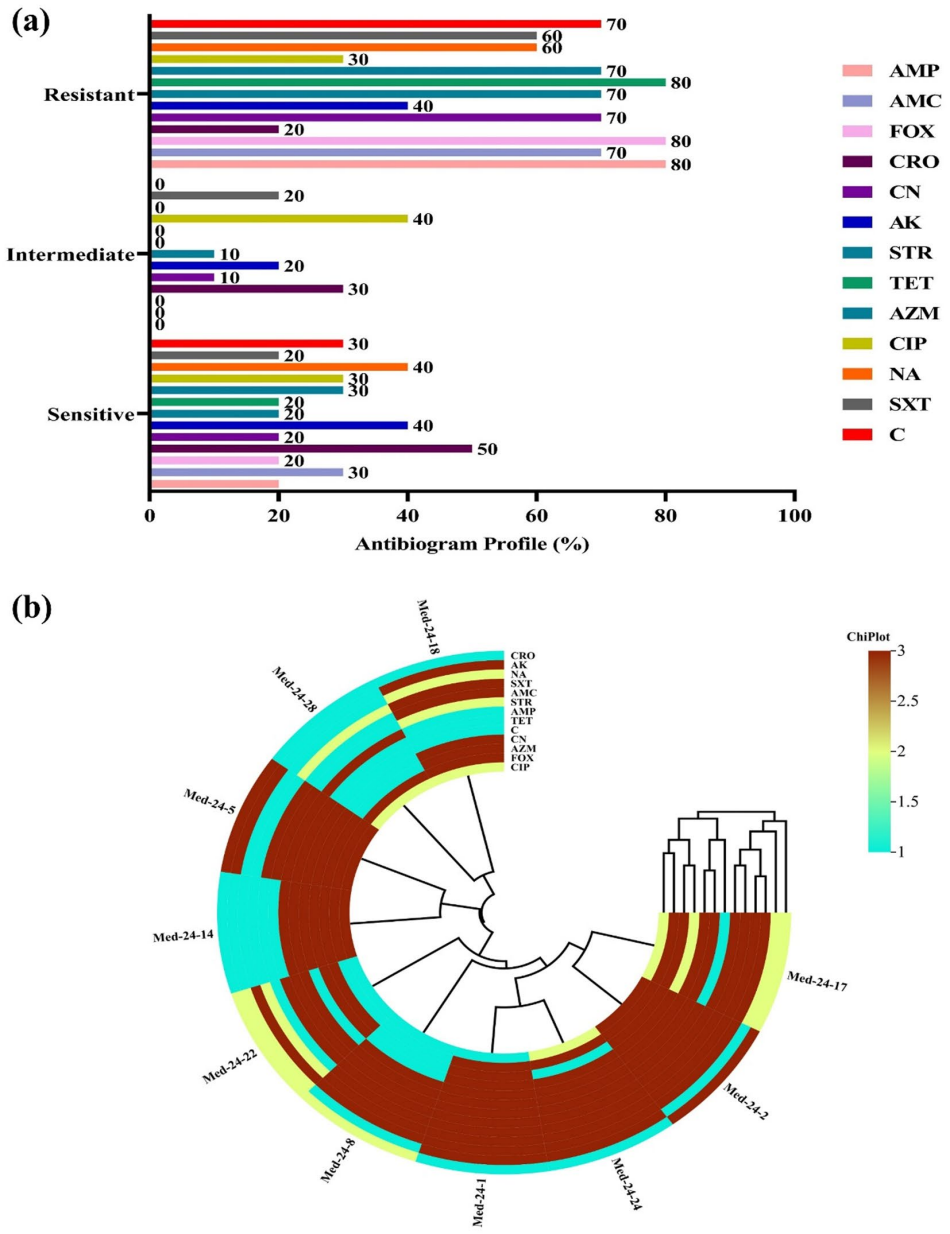
Antimicrobial resistance profile of MRSA isolates. (**a**) Sensitive, Intermediate and Resistant profile of MRSA isolates; (**b**) Polar Heat map showing resistance pattern the isolates.

The antibiogram profile of Methicillin-resistant *S. aureus* (MRSA) isolates revealed a high level of multidrug resistance. The isolates showed the highest resistance to ampicillin (80%), tetracycline (80%), and cefoxitin (80%), followed by azithromycin (70%), streptomycin (70%), gentamicin (70%), chloramphenicol (70%), and trimethoprim-sulfamethoxazole (60%). Intermediate resistance was observed against ciprofloxacin, ceftriaxone, and a few others. Among all tested antibiotics, the highest proportion of susceptible isolates was found for ceftriaxone (50%), followed by gentamicin (40%) and nalidixic acid (40%) shown in Fig. 4a.

The heatmap showed the antibiotic susceptibility patterns of MRSA isolates, revealing distinct clustering based on resistance profiles. Most isolates were resistant to commonly used antibiotics such as tetracycline, ampicillin, cefoxitin, and trimethoprim-sulfamethoxazole, showing widespread multidrug resistance. The dendrogram highlights similarities among isolates and antibiotic response patterns (Fig. 4b).

**Fig. 5:**
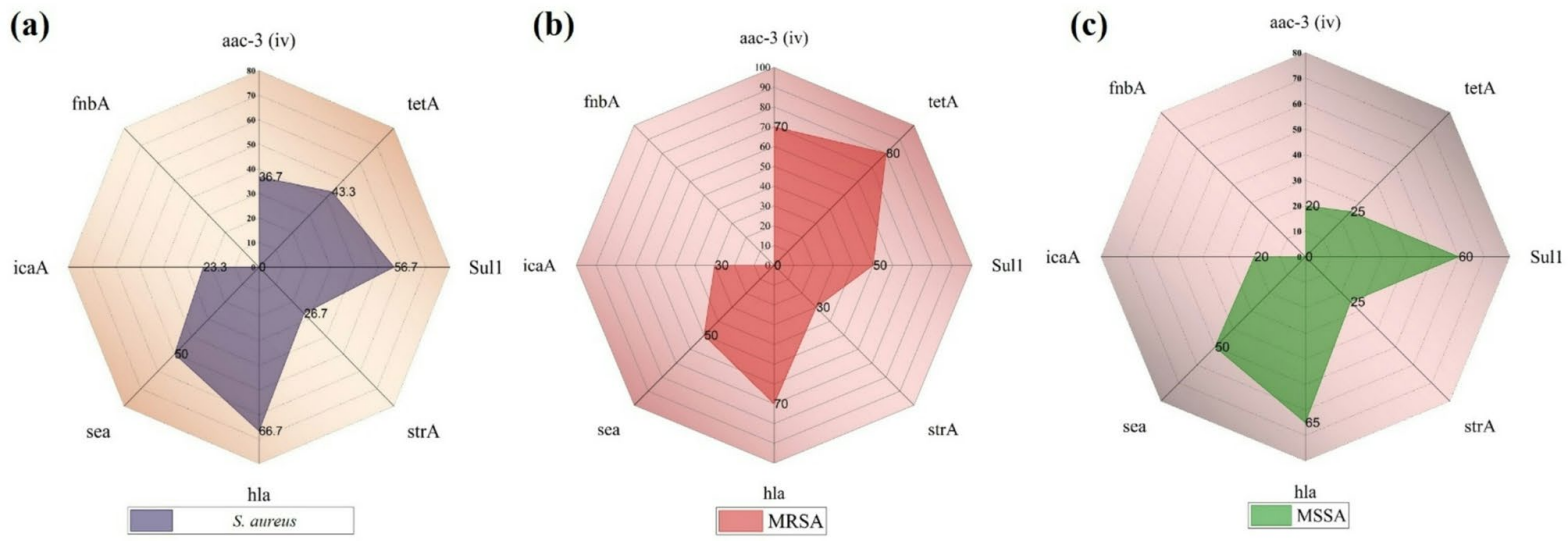
(**a**) Distribution of antimicrobial resistance and virulence genes among *S. aureus* (**b**) isolates, including MRSA and (**c**) MSSA.

Molecular analysis supported these findings, with key antimicrobial resistance genes and virulence genes detected at varying frequencies. The distribution of antimicrobial resistance and virulence genes among *Staphylococcus aureus* isolates, including MRSA and MSSA, showed considerable variation (Fig. 5). Among the 30 *S. aureus* isolates, resistance genes were detected at varying frequencies: *aac(3)-iv* in 36.7%, *tetA* in 43.3%, *sul1* in 56.7%, and *strA* in 26.7%. Notably, MRSA isolates harbored higher proportions of resistance genes, with *aac(3)-iv* (70.0%) and *tetA* (80.0%) being most prevalent (Table 5). In contrast, MSSA isolates showed lower frequencies for these genes: *aac(3)-iv* (20.0%) and *tetA* (25.0%). However, *sul1* was more common in MSSA (60.0%) than in MRSA (50.0%).

**Table 5:** Distribution of virulent and resistant genes isolated from MRSA (*n* = 10) isolates.

Habitat	*aac(3)-iv*	*tetA*	*sul1*	*strA*	*hla*	*sea*	*icaA*	*fnbA*
Bakshiganj (3)	2 (66.7)	3 (100.0)	2 (66.7)	2 (66.7)	1 (33.3)	2 (66.7)	0	0
Islampur (2)	2 (100.0)	1 (50.0)	1 (50.0)	0	2 (100.0)	1 (50.0)	2 (100.0)	0
Dewanganj (3)	1 (33.3)	3 (100.0)	1 (33.33)	0	3 (100.0)	2 (66.7)	0	0
Jamalpur (2)	2 (100.0)	1 (50.0)	1 (50.0)	1 (50.0)	1 (50.0)	0	1 (50.0)	0
Total (10)	7	8	5	3	7	5	3	0

Regarding virulence genes, *hla* was the most frequently detected (66.7%) among all *S. aureus* isolates, followed by *sea* (50.0%) and *icaA* (23.3%). MRSA and MSSA showed similar patterns for *hla* (70.0% vs. 65.0%) and *sea* (both 50.0%). The *icaA* gene was detected in 30.0% of MRSA and 20.0% of MSSA isolates. The *fnbA* gene was not detected in any of the isolates (Fig. 5).

### Resistance determinants of *S. aureus* and MRSA

Among the isolated *S. aureus* and MRSA strains, diverse antimicrobial resistance patterns were observed, with several isolates exhibiting XDR and MDR profiles. Among the *S. aureus* isolates (*n* = 30), 10% of *S. aureus* isolates showed resistance to 11 antibiotics across 8 classes, with a high MARI of 0.85, considered as XDR and also harboring *aac(3)-iv*,* tetA*, and *sul1* genes. Several other resistance patterns were also observed, with MARI values ranging from 0.38 to 0.77.

In the MRSA group (*n* = 10), a higher proportion (60%) of XDR patterns, with one isolate showing resistance to 12 antibiotics from 8 classes and the highest MARI (0.92). The ARGs frequently detected included *aac(3)-iv*,* tetA*,* sul1*, and *strA*, showed high resistance burden in MRSA compared to other *S. aureus* isolates. The results showed high burden of MDR and XDR in MRSA isolates (Table 6).

**Table 6:** Phenotypic antimicrobial resistance patterns, resistance types, ARGs, and MARI values of *S. aureus* and MRSA isolates.

Organisms	No. of isolates	Phenotypic pattern	Antibiotics (Class)	Resistance type	%	ARGs	MARI
*S aureus*	3	AMP-AMC-CN-AK-FOX-CRO-TET-NA-AZM-C- SXT	11 (8)	XDR	10.00	*aac(3)-iv*,* tetA*,* sul1*	0.85
	2	AMP-AMC-CN-AK-CRO-TET-AZM-C-SXT	9 (7)	XDR	6.67	*aac(3)-iv*,* tetA*,* sul1*	0.69
	2	AMP-AMC-CN-AK-CRO-TET-NA-AZM-C-SXT	10 (8)	XDR	6.67	*aac(3)-iv*,* tetA*,* sul1*	0.77
	2	AMP-AMC-CN-AK-STR-CRO-TET-NA-AZM-SXT	10(7)	XDR	6.67	*aac(3)-iv*,* tetA*,* sul1*,* strA*	0.77
	1	AMP-AK-CRO-NA-AZM	5(5)	MDR	3.33	*aac-3 (iv)*	0.38
	2	AMP-AMC-CN-AK-FOX-TET-NA-CIP	8 (5)	MDR	6.67	*aac(3)-iv*,* tetA*	0.62
	3	AMP-AMC-CN-AK-STR-FOX-CRO-TET-SXT	9 (5)	MDR	10.00	*aac(3)-iv*,* tetA*,* sul1*,* strA*	0.69
	1	AMP-AMC-CN-AK-STR-FOX-CRO-NA-CIP	9 (4)	MDR	3.33	*aac(3)-iv*,* strA*	0.69
	**16**				**53.34**		
MRSA	1	AMP-AMC-CN-STR-FOX-CRO-TET-NA-CIP-AZM-C-SXT	12(8)	XDR	10.00	*aac(3)-iv*,* tetA*,* sul1*,* strA*	0.92
	3	AMP-AMC-CN-STR-FOX-TET-AZM-C-SXT	9(7)	XDR	30.00	*aac(3)-iv*,* tetA*,* sul1*,* strA*	0.69
	2	AMP-AMC-CN-AK-STR-FOX-TET-NA-AZM-SXT	10(7)	XDR	20.00	*aac(3)-iv*,* tetA*,* sul1*,* strA*	0.69
	1	AMC-AK-STR-FOX-TET-NA-CIP-C	8(6)	MDR	10.00	*tetA*,* strA*	0.61
	1	AMP-CN-FOX-TET-NA-C	6(6)	MDR	10.00	*aac(3)-iv*,* tetA*,	0.46
	1	AMP-AK-CRO-NA-CIP-AZM-C	7 (6)	MDR	10.00	*aac(3)-iv*,* tetA*	0.61
	**9**				**90.00**		

### Biofilm production and its association with virulence and resistance genes

**Fig. 6:**
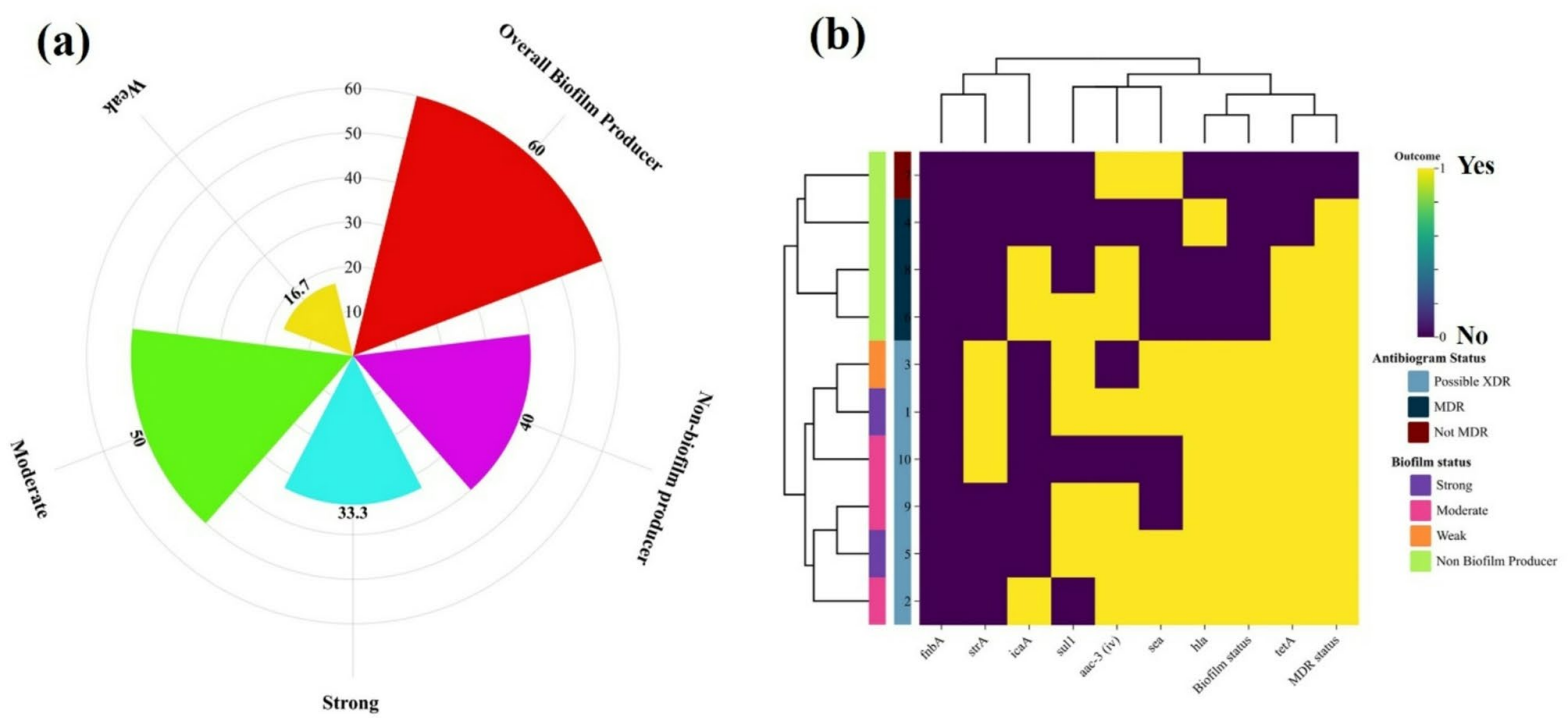
Biofilm production status among MRSA-positive isolates. (**a**) Classification of isolates based on biofilm-forming ability; (**b**) Heatmap illustrating the distribution and correlation between biofilm production and the presence of virulence and antimicrobial resistance genes.

Out of 10 MRSA-positive isolates, 6 (60%) demonstrated biofilm-forming ability (Fig. 6a). Among these biofilm-MRSA, 2 isolates (33.3%; 2/6) were strong biofilm producers, 3 (50%; 3/6) were moderate, and 1 (16.7%; 1/6) was a weak biofilm producer, while the remaining 4 isolates (40%) were non-biofilm producers. A heatmap was generated to visualize the association between biofilm formation, antimicrobial resistance (MDR/XDR) profiles, and the presence of virulence and resistance genes. Strong and moderate biofilm producers frequently co-expressed virulence genes such as *icaA*,* hla* and *sea*, along with resistance genes like *tetA*,* sul1*, and *aac(3)-iv.* The clustering pattern indicated a potential relationship between biofilm-forming ability and multidrug resistance, as most biofilm-producing isolates were categorized as MDR or XDR (Fig. 6b).

## Discussion

The present study provides critical insights into the prevalence, antibiotic resistance, and virulence attributes of *S. aureus*, particularly methicillin-resistant *S. aureus*, in subclinical mastitis cases among riverine buffaloes in Jamalpur, Bangladesh. The high quarter-level prevalence of SCM in different agro-ecological regions, indicating a substantial burden of intra-mammary infections in buffaloes under smallholder or semi-intensive systems.

The investigation revealed high prevalence in both animal and quarter level. The animal-level prevalence aligns closely with previously reported rates from Bangladesh (51.5%, 53.8%)^53,54^. However, it remains lower than the substantially higher prevalence figures documented in other studies from these regions, which range from 64.9 to 81.6%^35^. Conversely, the present findings exceed those from certain regions in India, where lower rates, such as 26.2%, have been reported^54^. Quarter-level prevalence in the current investigation (46.5%) mirrors observations from parts of India (45.8–61.6%) and Nepal (46.3%)^13,55^ yet stands considerably higher than figures reported in Bangladesh (27–42.5%)^33,52^ and markedly above the 19.3% recorded in Chitwan, Nepal^13^. Another study in Bangladesh showed a higher prevalence of SCM in buffaloes^47^. The apparently higher occurrence of subclinical mastitis (SCM) at the animal level than at the quarter level is largely a product of diagnostic protocols wherein the detection of one infected mammary quarter is sufficient to make the whole animal SCM positive^4^. Collectively, these reports underscore significant geographical differences in SCM prevalence, a process influenced by a diversity of interacting determinants, including local hygiene, milking procedures, lactation phase, and host genetic susceptibility. Adding to this variability are differences in geographical and climatic conditions, housing facilities, milking practices, udder hygiene, and the adoption of biosecurity practices. Moreover, differences in farmer awareness, education level, and access to veterinary care could further influence the risk profile of SCM.

The reported prevalence of subclinical mastitis (SCM) often varies depending on the diagnostic methods employed. While rapid screening tools such as the Modified Whiteside Test (MWST) and California Mastitis Test (CMT) provide quick results, they are generally less precise compared to molecular methods. Polymerase Chain Reaction (PCR), being highly sensitive, can detect low levels of bacterial DNA, often resulting in higher reported prevalence of SCM^56^. In the current study, conventional culture and biochemical analyses identified 22.4% of isolates as *Staphylococcus* spp., among which 21.2% were further confirmed by PCR. These findings differ from some previous reports from India and Bangladesh^24,55^. Specifically, the prevalence observed in this study is somewhat higher than that reported in a similar study conducted in Mymensingh, Bangladesh, yet lower than the rates documented in West Bengal, India^57,58^.

In this study, among the *Staphylococcus* spp. isolates, 41.1% were confirmed as *S. aureus*, which is comparable to the 37.4% reported in another study on riverine buffaloes in Bangladesh^24^. In this investigation, most of the isolates from our inquiry (58.9%) were non-aureus Staphylococci (NAS), similar to findings from previous studies performed in Bangladesh and Iran^56,59,60^. Because of their wide host range, adaptability (e.g., ability to form biofilm), and effective transmission, NAS may be more common. Diagnostic difficulties and antimicrobial resistance may also explain their presence. Moreover, among the *S. aureus* isolates, 33.3% were identified as methicillin-resistant (MRSA), closely matching findings from a similar study on riverine buffaloes in Bangladesh^24^. However, this prevalence exceeds that reported in Pakistan (19.6%)^61^ but remains lower than the 61.54% observed in a study from the Philippines^62^. Interestingly, the highest proportion of *S. aureus* isolates was detected in the right rear (RR) quarters, while MRSA was most frequently identified in the right front (RF) quarters (60.0%). This distribution contrasts with findings from Pakistan, where the left quarters were more frequently infected^61^. These differences reflect spatial heterogeneity of patterns of infection, potentially due to environmental stress or tension of a mechanical nature, that favors colonization of some udder quarters by *S. aureus* and MRSA.

The antibiogram profile and the heatmap analysis of MRSA isolates in this study indicated a significant level of multidrug resistance. Notably, the highest resistance rates were observed against tetracycline and cefoxitin. These findings are consistent with the findings of another study, which also documented high levels of resistance to cefoxitin and ampicillin in MRSA strains isolated in Egypt^63^. However, our data diverge from several earlier reports concerning the susceptibility patterns of tetracycline and trimethoprim-sulfamethoxazole. While these antibiotics have been described as effective treatment options against MRSA in previous studies^64,65^our results revealed high level of resistance (70–80%) to both agents. These differences may reflect geographical variations in antibiotic usage practices, selective pressure from over-the-counter availability, or clonal differences in MRSA strains across regions. Interestingly, ceftriaxone and amikacin demonstrated the highest susceptibility rates among the antibiotics tested in this investigation. This observation contrasts with numerous studies that identify vancomycin as the most effective and widely recommended therapeutic agent for MRSA infections^66,67^.

In this study, the *tetA* and *aac(3)-iv* resistance genes were considerably prevalent in *S. aureus* isolates, particularly in MRSA strains, indicating a strong association with multidrug resistance. This contrasts with findings from Pakistan, where *tetA* was common but *aac(3)-iv* was among the least prevalent^68^ suggesting regional differences in resistance gene dissemination. Additionally, the *sul1* gene was moderately prevalent across *S. aureus*, MRSA, and MSSA isolates, substantially lower than the 100% prevalence reported in Egypt^69^. These variations may reflect differences in local antibiotic usage and selective pressures. In this study, the *hla* gene was the most frequently detected virulence factor in *S. aureus* isolates (66.7%), followed by *icaA* and *sea*, while the *fnbA* gene was entirely absent. This pattern aligns with findings from Iraq, where *hla* was universally present (100%), and *icaA* and *sea* were also prevalent^70^, suggesting a conserved virulence profile in certain regional strains and a localized virulence signature potentially shaped by host–pathogen interactions, selective pressure, and ecological niche. However, the results contrast with a study from Egypt, where *sea* was the predominant gene (72.9%), followed by *icaA*, *hla*, and *fnbA*^71^. These differences likely reflect geographic and host-specific variations in *S. aureus* pathogenicity and also underscore the influence of regional ecological and epidemiological factors on virulence gene distribution^71^.

Furthermore, the study found that 53.3% of *Staphylococcus aureus* isolates exhibited multidrug resistance (MDR), a rate lower than that reported from clinical samples in Sudan but higher than the 39.4% MDR prevalence observed in animal-based food samples in Shanghai, China^72,73^. Additionally, 30% of isolates demonstrated XDR, a figure substantially exceeding previous reports, such as the 1.58% XDR prevalence in Sudanese clinical isolates^72^. This higher resistance may be driven by the bacteria’s capacity for horizontal gene transfer and its ecological adaptability, particularly in antibiotic-rich environments. Contributing factors likely include the misuse of antibiotics and insufficient infection control measures, which have been previously linked to the emergence and spread of XDR *S. aureus*^14^.

Variability in MDR and MARI values across studies likely reflects differences in antimicrobial usage patterns, sampling sources, geographic and temporal factors, and methodological approaches.

The emergence of biofilm-producing MRSA isolates presents a serious challenge in both veterinary and public health contexts. Biofilm formation significantly enhances bacterial survival by shielding the pathogens from host immune responses and antimicrobial treatments^74^. In this study, a substantial proportion of MRSA isolates from milk samples in Sylhet were identified as biofilm producers, with varying degrees of biofilm-forming ability. The coexistence of biofilm production with multiple virulence (e.g., *icaA*, *hla*,* sea*) and resistance genes (*tetA*, *sul1*, *aac(3)-iv*) suggests a synergistic relationship that likely contributes to enhanced pathogenicity and drug resistance^75,76^. Such isolates pose a significant threat, particularly when present in raw or improperly heated milk, potentially leading to foodborne infections^75^. The detection of strong biofilm producers with MDR/XDR profiles underlines the potential for these MRSA strains to act as superbugs. This situation necessitates the continuous monitoring of biofilm-associated traits due to their clinical and public health significance.

Although this study is limited by a relatively small sample size, restricted geographic coverage, and the absence of genotypic analysis of biofilm-associated genes, it highlights areas for future research to better understand the genetic linkage between biofilm formation, resistance, and virulence. Despite these limitations, the study’s strength lies in its integrated phenotypic and genotypic approach, offering critical insights into MRSA prevalence, antibiotic resistance, and virulence in subclinical mastitis of riverine buffaloes in Bangladesh. It effectively reveals the significant burden of MDR/XDR MRSA and its biofilm-forming potential, emphasizing serious risks to both animal and public health.

## Conclusion

This study clearly shows that subclinical mastitis is a major problem in buffaloes. *Staphylococcus aureus* was the main bacteria causing SCM, and 33.3% of them were methicillin-resistant. Many of the isolates were resistant to multiple antibiotics, with 60% being extensively drug-resistant, and the highest Multiple Antibiotic Resistance Index (MARI) was 0.92. Resistance genes like *tetA* (80%), *aac(3)-iv* (70%), and *sul1* (50%) were common. Virulence genes such as *hla* (66.7%), *sea* (50%), and *icaA* (23.3%) were also detected. Around 60% of the *S. aureus* isolates from raw milk could produce toxins and form biofilms. These findings show a serious risk to both animal health and human health, as these bacteria can spread from animals to people. These findings emphasize the urgent need for improved mastitis control strategies, rational antimicrobial use, and routine surveillance in buffalo farming systems. Moreover, enhancing awareness among farmers about subclinical mastitis, hygienic milking practices, and the consequences of indiscriminate antibiotic use is critical. This research provides important baseline data that can inform antimicrobial stewardship policies, guide targeted interventions, and contribute to the broader efforts to mitigate antimicrobial resistance in livestock and protect public health.

## Supplementary Information

Below is the link to the electronic supplementary material.

Supplementary Material 1

## Data Availability

The data supporting the findings of this study can be obtained from the corresponding authors upon reasonable request.

## References

[1] Hamid, M., Siddiky, M., Rahman, M. & Hossain, K. Scopes and opportunities of Buffalo farming in bangladesh: A review. *SAARC J. Agric.* **14**, 63–77 (2017).

[2] Chowdhury, M. S. et al. Subclinical mastitis of buffaloes in asia: prevalence, pathogenesis, risk factors, antimicrobial resistance, and current treatment strategies. *J. Anim. Sci. Technol.*10.5187/JAST.2024.E66 (2024). 10.5187/jast.2024.e66 PMC12923260 41726218

[3] Habib, M. R. et al. Dairy Buffalo production scenario in bangladesh: a review. *Asian J. Med. Biol. Res.* **3**, 305–316 (2017).

[4] Krishnamoorthy, P., Goudar, A. L., Suresh, K. P. & Roy, P. Global and countrywide prevalence of subclinical and clinical mastitis in dairy cattle and buffaloes by systematic review and meta-analysis. *Res. Vet. Sci.* **136**, 561–586 (2021). 33892366 10.1016/j.rvsc.2021.04.021

[5] DLS. Livestock Economy at a Glance (2023–2024). (2024). https://dls.portal.gov.bd/sites/default/files/files/dls.portal.gov.bd/page/ee5f4621_fa3a_40ac_8bd9_898fb8ee4700/2024-08-13-10-26-93cb11d540e3f853de9848587fa3c81e.pdf

[6] Salvador, R. T., Beltran, J. M. C., Abes, N. S., Gutierrez, C. A. & Mingala, C. N. Short communication: prevalence and risk factors of subclinical mastitis as determined by the California mastitis test in water buffaloes (Bubalis bubalis) in Nueva ecija, Philippines. *J. Dairy. Sci.* **95**, 1363–1366 (2012). 22365218 10.3168/jds.2011-4503

[7] Malik, M. H. & Verma, H. K. Prevalence, economic impact and risk factors associated with mastitis in dairy animals of Punjab. *Indian J. Anim. Sci.* **87**, 1452–1456 (2017).

[8] Singha, S. et al. Incidence, etiology, and risk factors of clinical mastitis in dairy cows under semi-tropical circumstances in chattogram, Bangladesh. *Animals* **11**, 2255 (2021). 34438713 10.3390/ani11082255 PMC8388477

[9] Singh, A. K. A comprehensive review on subclinical mastitis in dairy animals: pathogenesis, factors associated, prevalence, economic losses and management strategies. *CABI Reviews*. 10.1079/cabireviews202217057 (2022).

[10] Yildiz, S. C. & Yildiz, S. C. *Staphylococcus aureus* and methicillin resistant *Staphylococcus aureus* (MRSA) carriage and infections. (2022). 10.5772/INTECHOPEN.107138

[11] Costa, R. A., de Lira, J. V. & Aragão, M. F. Biofilm-formation by drug-resistant Staphylococcus aureus from cow milk. *J. Consum. Prot. Food Saf.* **14**, 63–69 (2019).

[12] Sharan, M., Dhaka, P., Bedi, J. S., Mehta, N. & Singh, R. Assessment of biofilm-forming capacity and multidrug resistance in Staphylococcus aureus isolates from animal-source foods: implications for lactic acid bacteria intervention. *Ann. Microbiol.* **74**, 1–14 (2024).

[13] Tiwari, B. B. et al. Prevalence and risk factors of Staphylococcal subclinical mastitis in dairy animals of chitwan, Nepal. *J. Pure Appl. Microbiol.* **16**, 1392–1403 (2022).

[14] Shrestha, A., Bhattarai, R. K., Luitel, H., Karki, S. & Basnet, H. B. Prevalence of methicillin-resistant Staphylococcus aureus and pattern of antimicrobial resistance in mastitis milk of cattle in chitwan, Nepal. *BMC Vet. Res* **17**, 239 (2021). 10.1186/s12917-021-02942-6 PMC8262063 34233667

[15] Algammal, A. M. et al. Methicillin-resistant Staphylococcus aureus (MRSA): one health perspective approach to the bacterium epidemiology, virulence factors, antibiotic-resistance, and zoonotic impact. *Infect. Drug Resist.* **13**, 3255–3265 (2020). 33061472 10.2147/IDR.S272733 PMC7519829

[16] Guccione, J. et al. Antibiotic dry Buffalo therapy: effect of intramammary administration of benzathine Cloxacillin against Staphylococcus aureus mastitis in dairy water Buffalo. *BMC Vet. Res* **16**, 191 (2020). 10.1186/s12917-020-02410-7 PMC7291747 32532337

[17] Hoque, M. N., Das, Z. C., Rahman, A. N. M. A., Haider, M. G. & Islam, M. A. Molecular characterization of Staphylococcus aureus strains in bovine mastitis milk in Bangladesh. *Int. J. Vet. Sci. Med.* **6**, 53–60 (2018). 30255079 10.1016/j.ijvsm.2018.03.008 PMC6147393

[18] Hoque, R. et al. Tackling antimicrobial resistance in bangladesh: A scoping review of policy and practice in human, animal and environment sectors. *PLoS One* **15**, e0227947 (2020). 10.1371/journal.pone.0227947 PMC6984725 31986167

[19] El-Ashker, M. et al. Staphylococci in cattle and buffaloes with mastitis in Dakahlia governorate, Egypt. *J. Dairy. Sci.* **98**, 7450–7459 (2015). 26364099 10.3168/jds.2015-9432

[20] Haran, K. P. et al. Prevalence and characterization of Staphylococcus aureus, including methicillin-resistant Staphylococcus aureus, isolated from bulk tank milk from Minnesota dairy farms. *J. Clin. Microbiol.* **50**, 688–695 (2012). 22170937 10.1128/JCM.05214-11 PMC3295154

[21] Hoque, M. N., Das, Z. C., Talukder, A. K., Alam, M. S. & Rahman, A. N. M. A. Different screening tests and milk somatic cell count for the prevalence of subclinical bovine mastitis in Bangladesh. *Trop. Anim. Health Prod.* **47**, 79–86 (2015). 25326717 10.1007/s11250-014-0688-0

[22] Farabi, A. et al. Prevalence, Risk factors, and antimicrobial resistance of Staphylococcus and Streptococcus species isolated from subclinical bovine mastitis. *Foodborne Pathog Dis* **22**, 467–476 (2024). 10.1089/fpd.2024.0097 39479784

[23] Petinaki, E. & Spiliopoulou, I. Methicillin-resistant Staphylococcus aureus among companion and food-chain animals: Impact of human contacts. *Clin. Microbiol. Infect.* vol. 18 626–634 Preprint at (2012). 10.1111/j.1469-0691.2012.03881.x 10.1111/j.1469-0691.2012.03881.x 22550956

[24] Hoque, M. N. et al. Antibiogram and virulence profiling reveals multidrug resistant *Staphylococcus aureus* as the predominant aetiology of subclinical mastitis in riverine buffaloes. *Vet. Med. Sci.* **8**, 2631–2645 (2022). 36136962 10.1002/vms3.942 PMC9677375

[25] Magiorakos, A. P. et al. Multidrug-resistant, extensively drug-resistant and pandrug-resistant bacteria: an international expert proposal for interim standard definitions for acquired resistance. *Clin. Microbiol. Infect.* **18**, 268–281 (2012). 21793988 10.1111/j.1469-0691.2011.03570.x

[26] Uddin, T. M. et al. Antibiotic resistance in microbes: History, mechanisms, therapeutic strategies and future prospects. *J. Infect. Public Health* vol. 14 1750–1766 Preprint at (2021). 10.1016/j.jiph.2021.10.020 10.1016/j.jiph.2021.10.020 34756812

[27] Salam, M. A. et al. Antimicrobial resistance: a growing serious threat for global public health. *Healthc. 2023*. **11**, 1946 (2023). 10.3390/healthcare11131946 PMC10340576 37444780

[28] Algammal, A. M. & Behzadi, P. Antimicrobial resistance: a global public health concern that needs perspective combating strategies and new talented antibiotics. *Discov Med.* **36**, 1911 (2024). 39327254 10.24976/Discov.Med.202436188.177

[29] Zhang, S. et al. Phenotypic and genotypic characterization of antimicrobial resistance profiles in Streptococcus dysgalactiae isolated from bovine clinical mastitis in 5 provinces of China. *J. Dairy. Sci.* **101**, 3344–3355 (2018). 29397161 10.3168/jds.2017-14031

[30] Ahangari, Z., Ghorbanpoor, M., Shapouri, M. R. S., Gharibi, D. & Ghazvini, K. Methicillin resistance and selective genetic determinants of Staphylococcus aureus isolates with bovine mastitis milk origin. *Iran. J. Microbiol.* **9**, 152–159 (2017). 29225754 PMC5719509

[31] Thrusfield, M. et al. Veterinary epidemiology: fourth edition. *Veterinary Epidemiology: Fourth Ed.* **1–861**10.1002/9781118280249 (2017).

[32] Naser, J. et al. Exploring of spectrum beta lactamase producing multidrug-resistant *Salmonella enterica* serovars in goat meat markets of Bangladesh. *Vet. Anim. Sci.* **25**, 100367 (2024). 38947184 10.1016/j.vas.2024.100367 PMC11214345

[33] Mia, M. P. et al. Prevalence and consequences of bovine subclinical mastitis in hill tract areas of the chattogram division, Bangladesh. *J. Adv. Biotechnol. Exp. Ther.* **8**, 163–181 (2025).

[34] NMC Protocols, guidelines and procedures - national mastitis council. (2016). https://www.nmconline.org/nmc-protocols-guidelines-and-procedures/

[35] Emon, A. et al. Prevalence, antimicrobial susceptibility profiles and resistant gene identification of bovine subclinical mastitis pathogens in Bangladesh. *Heliyon* **10**, e34567 (2024). 39816335 10.1016/j.heliyon.2024.e34567 PMC11734078

[36] Algammal, A. M., Enany, M. E., El-Tarabili, R. M., Ghobashy, M. O. I. & Helmy, Y. A. Prevalence, antimicrobial resistance profiles, virulence and enterotoxin-determinant genes of MRSA isolated from subclinical bovine mastitis samples in Egypt. *Pathogens* **9**, 362 (2020). 32397408 10.3390/pathogens9050362 PMC7281566

[37] Eidaroos, N. H. et al. Virulence traits, Agr typing, multidrug resistance patterns, and biofilm ability of MDR Staphylococcus aureus recovered from clinical and subclinical mastitis in dairy cows. *BMC Microbiol.* **25**, 1–16 (2025). 40102767 10.1186/s12866-025-03870-3 PMC11921537

[38] Smith, I. Veterinary microbiology and microbial disease. *Vet. J.* **165**, 333 (2003).

[39] Chowdhury, M. S. R. et al. Detection and prevalence of antimicrobial resistance genes in multidrug-resistant and extensively drug-resistant Staphylococcus and Streptococcus species isolated from Raw Buffalo milk in subclinical mastitis. *PLoS One*. **20**, e0324920 (2025). 40526704 10.1371/journal.pone.0324920 PMC12173402

[40] Aldous, W. K., Pounder, J. I., Cloud, J. L. & Woods, G. L. Comparison of six methods of extracting Mycobacterium tuberculosis DNA from processed sputum for testing by quantitative real-time PCR. *J. Clin. Microbiol.* **43**, 2471–2473 (2005). 15872286 10.1128/JCM.43.5.2471-2473.2005 PMC1153782

[41] Hwang, S. M., Kim, M. S., Park, K. U., Song, J. & Kim, E. C. Tuf gene sequence analysis has greater discriminatory power than 16S rRNA sequence analysis in identification of clinical isolates of coagulase-negative Staphylococci. *J. Clin. Microbiol.* **49**, 4142–4149 (2011). 21998419 10.1128/JCM.05213-11 PMC3232977

[42] McMurray, C. L., Hardy, K. J., Calus, S. T., Loman, N. J. & Hawkey, P. M. Staphylococcal species heterogeneity in the nasal Microbiome following antibiotic prophylaxis revealed by Tuf gene deep sequencing. *Microbiome* **4**, 63 (2016). 27912796 10.1186/s40168-016-0210-1 PMC5134057

[43] Harmer, C. J. & Hall, R. M. The A to Z of A/C plasmids. *Plasmid* **80**, 63–82 (2015). 25910948 10.1016/j.plasmid.2015.04.003

[44] Karimzadeh, R. & Ghassab, R. K. Identification of Nuc Nuclease and sea enterotoxin genes in Staphylococcus aureus isolates from nasal mucosa of burn hospital staff: a cross-sectional study. *New. Microbes New. Infect.* **47**, 100992 (2022). 35800028 10.1016/j.nmni.2022.100992 PMC9253491

[45] Freeman, D. J., Falkiner, F. R. & Keane, C. T. New method for detecting slime production by coagulase negative Staphylococci. *J. Clin. Pathol.* **42**, 872–874 (1989). 2475530 10.1136/jcp.42.8.872 PMC1142068

[46] Kouidhi, B., Zmantar, T., Hentati, H. & Bakhrouf, A. Cell surface hydrophobicity, biofilm formation, adhesives properties and molecular detection of adhesins genes in Staphylococcus aureus associated to dental caries. *Microb. Pathog*. **49**, 14–22 (2010). 20298773 10.1016/j.micpath.2010.03.007

[47] Chowdhury, M. S. R. et al. Emergence of highly virulent multidrug and extensively drug resistant Escherichia coli and Klebsiella pneumoniae in buffalo subclinical mastitis cases. *Sci. Rep. 2025 15:1* 15, 1–15 (2025). 10.1038/s41598-025-95914-x PMC11972387 40188167

[48] Golpasand, T., Keshvari, M. & Behzadi, P. Distribution of chaperone-usher fimbriae and curli fimbriae among uropathogenic Escherichia coli. *BMC Microbiol* **24**, 344 (2024). 10.1186/s12866-024-03472-5 PMC11401301 39271999

[49] CLSI. CLSI M100-ED33: 2023 Performance standards for antimicrobial susceptibility testing, 33rd Edition. Clsi 402. (2023).

[50] Roy, M. C. et al. Zoonotic linkage and environmental contamination of Methicillin-resistant Staphylococcus aureus (MRSA) in dairy farms: A one health perspective. *One Health*. **18**, 100680 (2024). 39010963 10.1016/j.onehlt.2024.100680 PMC11247269

[51] Cosentino, F., Viale, P. & Giannella, M. MDR/XDR/PDR or DTR? Which definition best fits the resistance profile of Pseudomonas aeruginosa? *Curr. Opin. Infect. Dis.* **36**, 564–571 (2023). 37930070 10.1097/QCO.0000000000000966 PMC10836784

[52] Singha, S. et al. The prevalence and risk factors of subclinical mastitis in water Buffalo (Bubalis bubalis) in Bangladesh. *Res. Vet. Sci.* **158**, 17–25 (2023). 36907020 10.1016/j.rvsc.2023.03.004

[53] Islam, J. et al. Assessment of subclinical mastitis in Milch animals by different field diagnostic tests in Barishal district of Bangladesh. *Asian-Australasian J. Biosci. Biotechnol.* **4**, 24–33 (2019).

[54] Srinivasan, P. et al. Prevalence and etiology of subclinical mastitis among buffaloes (Bubalus bubalus) in Namakkal, India. *Pak. J. Biol. Sci.* vol. 16 1776–1780 Preprint at (2013). 10.3923/pjbs.2013.1776.1780 10.3923/pjbs.2013.1776.1780 24506047

[55] Preethirani, P. L. et al. Isolation, biochemical and molecular identification, and in-vitro antimicrobial resistance patterns of bacteria isolated from Bubaline subclinical mastitis in South India. *PLoS One*. **10**, e0142717 (2015). 26588070 10.1371/journal.pone.0142717 PMC4654528

[56] Singha, S. et al. Occurrence and aetiology of subclinical mastitis in water Buffalo in Bangladesh. *J. Dairy. Res.* **88**, 314–320 (2021). 34412714 10.1017/S0022029921000698

[57] Karmakar, A., Dua, P. & Ghosh, C. Biochemical and molecular analysis of staphylococcus aureus clinical isolates from hospitalized patients. *Can. J. Infect. Dis. Med. Microbiol.* 9041636 (2016). (2016). 10.1155/2016/9041636 PMC4904573 27366185

[58] Urmi, M. R. et al. Antibiotic resistance patterns of Staphylococcus spp. Isolated from fast foods sold in different restaurants of mymensingh, Bangladesh. *J. Adv. Vet. Anim. Res.* **8**, 274 (2021). 34395598 10.5455/javar.2021.h512 PMC8280991

[59] Beheshti, R. et al. Prevalence and etiology of subclinical mastitis in Buffalo of the Tabriz region. *Iran. J. Am. Sci.* **7**, 1545–1003 (2011).

[60] Paul, O. B. Prevalence of subclinical mastitis and associations with risk factors in buffaloes in Noakhali district of Bangladesh. Thesis-MS. http://dspace.cvasu.ac.bd/jspui/handle/123456789/1631 (2021).

[61] Javed, S. et al. Epidemiology and molecular characterization of Staphylococcus aureus causing bovine mastitis in water buffaloes from the Hazara division of Khyber pakhtunkhwa, Pakistan. *PLoS One*. **17**, e0268152 (2022). 35512008 10.1371/journal.pone.0268152 PMC9071125

[62] Badua, A. T., Boonyayatra, S., Awaiwanont, N., Gaban, P. B. V. & Mingala, C. N. Methicillin-resistant Staphylococcus aureus (MRSA) associated with mastitis among water buffaloes in the Philippines. *Heliyon* **6**, e05663 (2020). 33319108 10.1016/j.heliyon.2020.e05663 PMC7723804

[63] Alfeky, A. A. E., Tawfick, M. M., Ashour, M. S. & El-Moghazy, A. N. A. High prevalence of multi-drug resistant methicillin-resistant Staphylococcus aureus in tertiary Egyptian hospitals. *J. Infect. Dev. Ctries.* **16**, 795–806 (2022). 35656950 10.3855/jidc.15833

[64] Akanbi, O. E., Njom, H. A., Fri, J., Otigbu, A. C. & Clarke, A. M. Antimicrobial susceptibility of Staphylococcus aureus isolated from recreational waters and beach sand in Eastern cape Province of South Africa. *Int J. Environ. Res. Public. Health* **14**, 1001 (2017). 10.3390/ijerph14091001 PMC5615538 28862669

[65] Tălăpan, D., Sandu, A. M. & Rafila, A. Antimicrobial resistance of Staphylococcus aureus isolated between 2017 and 2022 from infections at a tertiary care hospital in Romania. *Antibiotics (Basel)* **12**, 974 (2023). 10.3390/antibiotics12060974 PMC10294969 37370293

[66] Al-Sarar, D., Moussa, I. M. & Alhetheel, A. Antibiotic susceptibility of methicillin-resistant Staphylococcus aureus (MRSA) strains isolated at tertiary care hospital in riyadh, Saudi Arabia. *Medicine* **103**, E37860 (2024). 38640320 10.1097/MD.0000000000037860 PMC11029994

[67] Wang, W. et al. High-level Tetracycline resistance mediated by efflux pumps Tet(A) and Tet(A)-1 with two start codons. *J. Med. Microbiol.* **63**, 1454–1459 (2014). 25102906 10.1099/jmm.0.078063-0

[68] Khan, S. B. et al. Prevalence of antibiotic resistant genes in Staphylococcus aureus isolated from bovine mastitis. *Pak J. Zool.* **54**, 2239–2244 (2022).

[69] Tarabees, R. Molecular characterization of Staphylococcus species isolated from clinical and subclinical mastitic buffaloes in el-behera governorate, Egypt. *Alex J. Vet. Sci.* **69**, 20–20 (2021).

[70] Abase, H. M. & Abdalhadi Hussain, E. Detection of meca, icaa, hlα, sea genes, and histological changes in mice for Staphylococcus aureus isolated from vaginosis in Iraqi women. *Basrah Res. Sci.* **50**, 9–19 (2024).

[71] Rasmi, A. H., Ahmed, E. F., Darwish, A. M. A. & Gad, G. F. M. Virulence genes distributed among Staphylococcus aureus causing wound infections and their correlation to antibiotic resistance. *BMC Infect. Dis.* **22**, 1–12 (2022). 35902813 10.1186/s12879-022-07624-8 PMC9547454

[72] Moglad, E. H. & Altayb, H. N. Antibiogram, prevalence of methicillin-resistant and multi-drug resistant Staphylococcus spp. In different clinical samples. *Saudi J. Biol. Sci* **29**, 103432 (2022). 10.1016/j.sjbs.2022.103432 PMC9478920 36117784

[73] Ou, C. et al. Prevalence of multidrug-resistant Staphylococcus aureus isolates with strong biofilm formation ability among animal-based food in Shanghai. *Food Control*. **112**, 107106 (2020).

[74] Rather, M. A., Gupta, K. & Mandal, M. Microbial biofilm: formation, architecture, antibiotic resistance, and control strategies. *Brazilian J. Microbiol.* **52**, 1701–1718 (2021). 10.1007/s42770-021-00624-x PMC8578483 34558029

[75] Kamali, E., Jamali, A., Ardebili, A., Ezadi, F. & Mohebbi, A. Evaluation of antimicrobial resistance, biofilm forming potential, and the presence of biofilm-related genes among clinical isolates of Pseudomonas aeruginosa. *BMC Res. Notes*. **13**, 1–6 (2020). 31924268 10.1186/s13104-020-4890-z PMC6954586

[76] Behzadi, P. et al. Relationship between biofilm-formation, phenotypic virulence factors and antibiotic resistance in environmental *Pseudomonas aeruginosa*. *Pathogens* **11**, 1015 (2022). 36145447 10.3390/pathogens11091015 PMC9503712

[77] Kozak, G. K., Boerlin, P., Janecko, N., Reid-Smith, R. J. & Jardine, C. Antimicrobial resistance in *Escherichia coli* isolates from swine and wild small mammals in the proximity of swine farms and in natural environments in ontario, Canada. *Appl. Environ. Microbiol.* **75**, 559–566 (2009). 19047381 10.1128/AEM.01821-08 PMC2632148

