# Correction: Biofilm production and virulence traits among extensively drug-resistant and methicillin-resistant *Staphylococcus aureus* from buffalo subclinical mastitis in Bangladesh

**DOI:** 10.1038/s41598-025-28806-9

**Published:** 2025-12-04

**Authors:** Md Shahidur Rahman Chowdhury, Md Mosharof Hosen, Hemayet Hossain, Md Rafiqul Islam, Md Bashir Uddin, Md Masudur Rahman, Md Mukter Hossain, Md Mahfujur Rahman

**Affiliations:** 1 Department of Medicine, Sylhet Agricultural University, Sylhet, 3100 Bangladesh; 2 Department of Anatomy and Histology, Sylhet Agricultural University, Sylhet, 3100 Bangladesh; 3 Department of Pathology, Sylhet Agricultural University, Sylhet, 3100 Bangladesh

Correction to: *Scientific Reports* 10.1038/s41598-025-17476-2, published online 02 October 2025

The original version of this Article contained errors in the names of the authors Md Shahidur Rahman Chowdhury, Md Mosharof Hosen, Md Rafiqul Islam, Md Bashir Uddin, Md Masudur Rahman, Md Mukter Hossain and Md Mahfujur Rahman, which were incorrectly given as Shahidur Rahman Chowdhury, Mosharof Hosen, Rafiqul Islam, Bashir Uddin, Masudur Rahman, Mukter Hossain and Mahfujur Rahman.

The original Article has been corrected.

