# Supplementary material for: Biofilm production and virulence traits among extensively drug-resistant and methicillin-resistant Staphylococcus aureus from buffalo subclinical mastitis in Bangladesh
Source: Sci Rep. 2025 Oct 2;15:34425. doi: 10.1038/s41598-025-17476-2 (PMC12491446; doi:10.1038/s41598-025-17476-2)
Supplement: Supplementary file 1 — Supplementary Material 1 [file 41598_2025_17476_MOESM1_ESM.docx]

**Supplementary Table 1:** Frequencies of Antimicrobial Resistant and virulent genes among *S. aureus*, MRSA and MSSA

| **Type** | **Genes Name** | ***S aureus* (n=30, 41.1%)** | **MRSA (n=10, 33.33%)** | **MSSA (n=20, 66.67%)** |
| --- | --- | --- | --- | --- |
| *Resistant gene* | *aac-3(iv)* | 11(36.67) | 7(70.0) | 4(20.0) |
|  | *tetA* | 13(43.33) | 8(80.0) | 5(25.0) |
|  | *Sul1* | 17(56.67) | 5(50.0) | 12(60.0) |
|  | *strA* | 8(26.67) | 3(30.0) | 5(25.0) |
|  |  |  |  |  |
| *Virulence genes* | *hla* | 20(66.67) | 7(70.0) | 13(65.0) |
|  | *sea* | 15(50.0) | 5(50.0) | 10(50.0) |
|  | *icaA* | 7(23.33) | 3(30.0) | 4(20.0) |
|  | *fnbA* | 0 | 0 | 0 |

**Supplementary Table 2:** Break point and disc potency of the selected antibiotics of this study based on CLSI 2020.

| **Antimicrobial class** | **Antibiotics** | **Disc potency (ug)** | **Interpretation** | | |
| --- | --- | --- | --- | --- | --- |
|  |  |  | **Sensitive (mm)** | **Intermediate**  **(mm)** | **Resistant**  **(mm)** |
| Penicillin | Ampicillin (AMP) | 10 | ≥29 | --- | ≤28 |
|  | Amoxicillin/  Clavulanic acid (AMC) | 20/10 | ≥29 | --- | ≥28 |
| Cephems including Cephalosporins | Cefoxitin (FOX) | 30 | ≥22 | --- | ≥21 |
|  | Ceftriaxone (CRO) | 30 | ≥23 | 20-22 | ≥19 |
| Aminoglycosides | Gentamicin (CN) | 10 | ≥15 | 13-14 | ≥12 |
|  | Amikacin (AK) | 30 | ≥20 | 17-19 | ≥16 |
|  | Streptomycin (STR) | 10 | ≥15 | 12-14 | ≥11 |
| Tetracyclines | Tetracycline (TET) | 30 | ≥19 | 15-18 | ≥14 |
| Macrolides | Azithromycin (AZM) | 15 | ≥18 | 14-17 | ≥13 |
| Quinolones | Ciprofloxacin (CIP) | 5 | ≥21 | 16-20 | ≥15 |
|  | Nalidixic acid (NA) | 30 | ≥19 | 14-18 | ≥13 |
| Folate pathway antagonists | Trimethoprim- Sulfamethoxazole (SXT) | 1.25/23.75 | ≥16 | 11-15 | ≥10 |
| Phenicol | Chloramphenicol (C) | 30 | ≥18 | 13-17 | ≥12 |
